# A PSO-based multi-objective multi-label feature selection method in classification

**DOI:** 10.1038/s41598-017-00416-0

**Published:** 2017-03-23

**Authors:** Yong Zhang, Dun-wei Gong, Xiao-yan Sun, Yi-nan Guo

**Affiliations:** School of Information and Electronic Engineering, China University of Mining and Technology, Xunzhou, 221116 China

## Abstract

Feature selection is an important data preprocessing technique in multi-label classification. Although a large number of studies have been proposed to tackle feature selection problem, there are a few cases for multi-label data. This paper studies a multi-label feature selection algorithm using an improved multi-objective particle swarm optimization (PSO), with the purpose of searching for a Pareto set of non-dominated solutions (feature subsets). Two new operators are employed to improve the performance of the proposed PSO-based algorithm. One operator is adaptive uniform mutation with action range varying over time, which is used to extend the exploration capability of the swarm; another is a local learning strategy, which is designed to exploit the areas with sparse solutions in the search space. Moreover, the idea of the archive, and the crowding distance are applied to PSO for finding the Pareto set. Finally, experiments verify that the proposed algorithm is a useful approach of feature selection for multi-label classification problem.

## Introduction

Multi-label feature selection (MFS) exists widely in engineering practice, such as image processing^1, 2^, and text categorization^3^. Its purpose is to remove irrelevant/redundant features, which is able to decrease the complexity of classifier, even improve the classification performance^4^. Since each sample is associated with multiple labels simultaneously and those labels are not mutually exclusive, this problem is much more difficult than traditional single-label feature selection. Classical method is to transform a multi-label problem into a traditional single-label one^5–8^. However, the kind of method is often inefficient for solving multi-label problems, because a new created label often contain many classes, resulting in the decreases of learning performance. On the other hand, feature selection is challenging in nature, since the search space of an algorithm increases exponentially with the number of available features^9, 10^.

Due to well global search capability, evolutionary algorithms (EAs) have been widely used solve feature selection in the single-label case. Part work includes genetic algorithm (GA)^11, 12^, ant colony optimization algorithm (ACO)^13^, and differential evolution algorithm (DE)^14^. As a relatively new EA algorithm, the PSO algorithm shows many advantages (such as simple implement and fast convergence)^15^. Thus, it has also been applied to feature selection in recent years^9, 16–19^.

However, few researches in those literatures have focused on the application of EAs in multi-label feature selection. Zhang *et al*. proposed a GA-based multi-label feature selection algorithm by using the accuracy of a multi-label classifier to estimate the fitness of feature subset^20^. Yu and Wang proposed a supervised feature selection algorithm for a multi-label data set based on mutual information and GAs^21^. The first step of this algorithm employs mutual information to fulfill local feature selection. Based on the result of local feature selection, GA is then adopted to select the global optimal feature subset at the two stages. However, since GA often costs much time in seeking a feature subset, these GA-based algorithms have the disadvantage of premature converges. To overcome this drawback, Lee and Kim presented a memetic-based multi-label feature selection algorithm^22^. In this algorithm, after a feature subset is found by using genetic search, a memetic process is employed to refine this subset further. In our recent work, we developed a DE-based multi-label feature selection algorithm^23^. However, the performance of that algorithm is not compared with any other EA-based algorithms. Recently, Pereira *et al*. provided a review of nature-inspire multi-label feature selection approaches^24^.

Actually, MFS is a kind of multi-objective optimization problems, which includes at least two conflicting objectives, i.e., maximizing the classification accuracy and minimizing the size of feature subset. In this paper we study a multi-objective optimization approach for MFS, for finding a set of feature subsets (solutions) to meet different requirements of decision-makers. Focused on this goal, an improved multi-objective PSO algorithm is developed by employing the probability-based encoding operator, the adaptive uniform mutation, the local learning strategy, and the archiving method based on crowding distance.

## Particle swarm optimization

As a population-based search method, PSO regards each individual in the population as a particles in search space. Supposing the location of the *i*-th particle is ${P}_{i}(t)=({p}_{i,1},{p}_{i,2},\cdots ,{p}_{i,D})$, its velocity is ${V}_{i}(t)=({v}_{i,1},{v}_{i,2},\cdots ,{v}_{i,D})$, the optimal location found by this particle so far (i.e., the local best position, Lbest) is $L{b}_{i}(t)=(l{b}_{i,1},l{b}_{i,2},\cdots ,l{b}_{i,D})$, the optimal location found by the swarm so far (i.e., the global best position, Gbest) is $G{b}_{i}(t)=(g{b}_{i,1},g{b}_{i,2},\cdots ,g{b}_{i,D})$, then, this particle is updated as follows^15^:

1 $$\{\begin{array}{rcl}{v}_{i,j}(t+1) & = & w\times {v}_{i,j}(t)+{r}_{1}\times {c}_{1}\times (l{b}_{i,j}(t)-{p}_{i,j}(t))+{r}_{2}\times {c}_{2}\times (g{b}_{i,j}(t)-{p}_{i,j}(t))\\ {p}_{i,j}(t+1) & = & {p}_{i,j}(t)+{v}_{i,j}(t+1).\end{array}$$

where, *t* is the iteration times, *c*_1_ and *c*_2_ are two acceleration coefficients, *r*_1_ and *r*_2_ are random numbers between [0, 1], and *w* is an inertia weight of particle on fly velocity.

## Methods

This section shows the PSO-based multi-objective multi-label feature selection algorithm. First a probability-based encoding operator instead of traditional binary encoding is employed to represent a particle. Based on this, a discrete multi-label feature selection problem is transformed into a continuous one suitable for the PSO. Second, we give the fitness function of multi-label feature selection problem, and introduce an archive to save optimal solutions obtained by the swarm. Next, an adaptive uniform mutation with action range varying over time, and a local learning strategy are proposed. Finally the implementing steps and the computational complexity of the algorithm are discussed.

### Encoding and Fitness function of Particle

In order to transform a discrete multi-label feature selection problem into a continuous one suitable for PSO, this paper employs a real encoding strategy, called the probability-based encoding strategy^4^. This method takes the probability value that a feature is selected as an encoding element of particle. Thus a particle including a number of probability values is a candidate solution of the problem. Taking a particle ${P}_{i}(t)=({p}_{i,1},{p}_{i,2},\cdots ,{p}_{i,D})$ as an example, if the probability ${p}_{i,d} > 0.5,d=1,2,\cdots D,$ then the *d*-th feature is chosen into the corresponding feature subset; otherwise it is not.

This paper adopts two objectives, the multi-label classification error and the number of features, as the fitness function of the algorithm. Various measures have been designed to evaluate the classification performance of a multi-label classifier, including Hamming loss, accuracy, one-error, coverage, ranking loss, and so on ref. 25. Like some multi-label classification methods^2, 5, 26^, this paper uses Hamming loss (*Hloss*) to evaluate the classification error rate of a particle. Let |*S*| be the number of samples in test dataset, *S*, the set of true class labels and that of labels predicted by a classifier, *h*, be *y*_*i*_ and ${y}_{i}^{^{\prime} },i=1,2,\cdots ,|S|$, respectively, the Hamming loss is defined as follows:

2 $$Hloss(h,S)=\frac{1}{|S|}\sum _{i=1}^{|S|}\frac{1}{|C|}|{y}_{i}{\rm{\Delta }}{y}_{i}^{^{\prime} }|$$

where Δ represents the symmetric difference between two sets, and |*C*| means the number of labels. Thus, the fitness function of a particle is described as:

3 $${\rm{\min }}\,F({P}_{i})=(Hloss({P}_{i},S),|{P}_{i}|)$$

where |*Pi*| is the number of features within the particle *P*_*i*_.

### Adaptive uniform mutation

**Figure 1:**
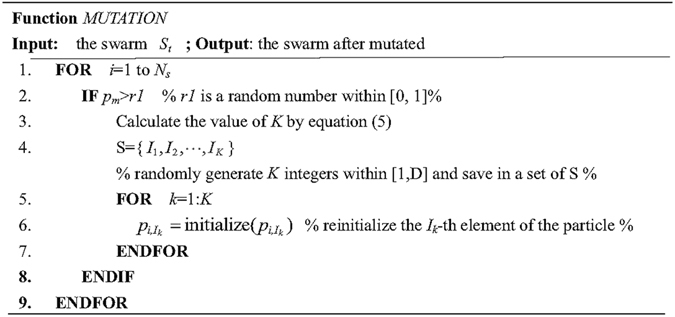
The pseudocode of the function MUTATION.

PSO is known to have a fast convergence speed. However, fast convergence speed often makes a PSO-based algorithm converge to a false Pareto front^27, 28^. In the paper, an adaptive uniform mutation is employed to extend the ability of the proposed algorithm in exploration. The details of the proposed mutation are given in Fig. 1. In this operator, a nonlinear function, *p*_*m*_, in terms of iterations, is adopted to control both the probability and range of mutation on each particle. At each iteration, first, *p*_*m*_ is updated according to the following approach:

4 $${p}_{m}=0.5\ast {e}^{(-10\ast t/T)}+0.01$$

where *T* is the maximum iteration times. It can be seen that the value of *p*_*m*_ tends to decrease at an exponential rate as the iterations increases. Then, each particle in the swarm is checked in turn. If *p*_*m*_ is bigger than a random number between [0, 1], we run the mutation on the current particle as follows: first pick randomly *K* elements from this particle, and then re-initialize the values of these elements within the search space. Here the value of *K* is an integer which is used to control the mutation range:

5 $$K=\,{\rm{\max }}\{1,\lceil D\ast {p}_{m}\rceil \}$$

On the one hand, this operator can show a good explorative behavior at the beginning of the algorithm, because nearly half of the particles in the swarm are affected by the operator with a high mutation space; on the other hand, it can improve the exploitative behavior of the swarm at the second half of the algorithm, because both the probability and range of the mutation decrease simultaneously with the increasing of iteration times.

### Local learning strategy

To improve the performance of the proposed algorithm, especially self-learning capability of elite particle in the swarm, a local research strategy based on differential learning is designed to explore the areas with sparse solutions in search space. In this strategy, first a solution with big crowding distance in the archive is selected as a base vector, notified *X*_*best*_, in differential learning. Second, two random solutions from the archive, notified *X*_*n*1_ and *X*_*n*2_, are set as differential vectors. Then, a new solution is generated by adding the difference between *X*_*n*1_ and *X*_*n*2_ to the base vector *X*_*best*_:

6 $${X}_{i}^{^{\prime} }={X}_{best}+F\cdot ({X}_{n1}-{X}_{n2})$$

This loop is implemented repeatedly until generating *N*′ new solutions. Finally, the *N*′ new solutions are saved into the archive. The parameter *F* is a scale factor that amplifies the difference between the two vectors. This paper sets *F* to be a random value within [0.1, 0.9] in order to improve the diversity of new solutions. Since the base vector often locates to good promising area, the local research strategy is competent for exploiting the area including sparse solutions.

### Implement of the algorithm

This section shows implementing steps of the proposed PSO-based algorithm:

**Step 1:** Initialize a swarm of particles. (a) Set the size of swarm, *N*_s_, the size of archive, *N*_*a*_, and the maximal number of iterations, *T*; (b) Initialize the locations of particles; (c) Evaluate all objectives of each particle; (d) Save non-dominated solutions into the archive.

**Step 2:** Update the personal best positions for particles. This paper uses the Pareto domination relationship to update the personal best positions. Taking a particle *P*_*i*_(*t*) as an example, if its new position *P*_*i*_(*t* + 1) dominates the old personal best position *Lb*_*i*_(*t*), set *Lb*_*i*_(*t* + 1) = *P*_*i*_(*t* + 1); otherwise, keep the personal best position in memory unchanged.

**Step 3:** Update the global best position of each particle. For each particle, we select the global best position from the archive based on the diversity of solutions. First, the crowding distance value of each solution in the archive is calculated. Then, based on the crowding distances above, the binary tournament is employed to select the global best position for the current particle. For any solution in the archive, the bigger its crowding distance is, the more the probability that it is selected as the global best position is.

**Step 4:** Generate new positions for each particle. For each particle, the equation (1) is employed to update its velocity and position. Different from most PSO-based algorithms with fixed control parameters, we set the two acceleration coefficients, c_1_ and c_2_, to linear functions over the number of iterations, respectively^29^.

**Step 5:** Perform the proposed uniform mutation above.

**Step 6:** Evaluate all objectives of each particle based on equation (3).

**Step 7:** Update the external archive. First, save all the new particles that don’t dominated by other solutions into the archive; if the number of solutions saved into the archive reaches its maximal capacity *N*_*a*_, the |*Ar*| − *N*_*a*_ solutions with worst distribution are deleted from the archive, where |*Ar*| is the number of solutions saved in the archive. In this paper, the crowding distance method^30^ is introduced to evaluate the distribution of solutions among the archive, since this technology does not involve parameters.

**Step 8:** Implement the local learning strategy above.

**Step 9:** Check the termination condition. If the algorithm meets the maximal number of iterations, *T*, stop and output the final solutions; otherwise, return Step 2.

**Figure 2:**
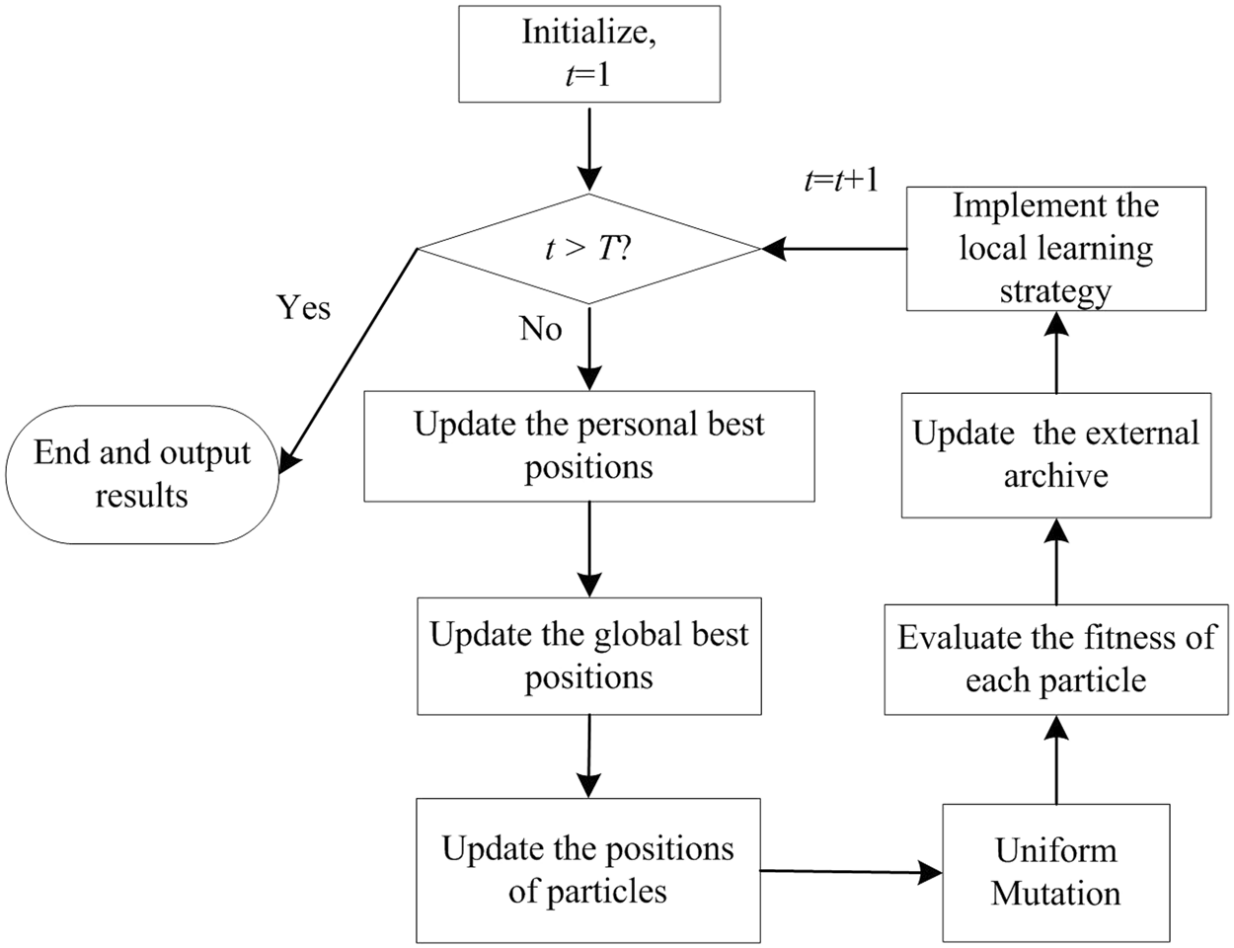
The flowchart of the proposed algorithm.

Furthermore, the flowchart of the proposed algorithm is showed in Fig. 2.

### Complexity Analysis

In the proposed algorithm, Step 1, 4, 5, 6 and 9 need O(1) basic comparison operation. In Step 2 and 3, the update of Pbest and Gbest need O(*M* × *N*_s_) and O(*N*_s_) basic operations, respectively. The computation complexity of the proposed algorithm lies mainly in Steps 7 and 8. In Step 7, the Pareto domination comparison costs O(*M* × *N* × log *N*) basic operations, and the crowding distance measure costs O(*M* × *N*_a_ × log *N*_a_) operations. In Step 8, both the Pareto domination comparison and the crowding distance measure need O(*M* × *N*_a_) operations. Owing to *N* = *N*_*s*_ + *N*_*a*_, in the worst case the computation complexity of our algorithm is simplified as O(*M* × *N* × log *N*).

However, when the proposed algorithm applies to a real feature selection problem, it's hard to calculate its real run-time. Like other EA-based algorithms, the run-time of our algorithm lies mainly in evaluating the fitness function. The evaluation time of a particle depends on the number of features, which is hard to predict. So, the run-time of the proposed algorithm depends on both the algorithm and the data sets.

## Results and Analyses

The performance of our proposed algorithms is discussed on six datasets from various applications, such as image processing, bioinformatics, music emotion, and so on. These datasets includes Emotions, Yeast, Scene, Flags, CAL500 and Birds. The format of these data sets is listed in Table 1, which includes the number of training samples, testing samples, features, and labels. These data sets above are freely available at the website of Mulan (http://mulan.sourceforge.net/datasets.html).

**Table 1:** Format of six datasets.

Data sets	Number of training samples	Number of testing samples	Number of labels	Number of features
Flags	129	65	7	19
CAL500	250	252	174	68
Emotions	391	202	6	72
Yeast	1500	917	14	103
Birds	322	323	19	260
Scene	1211	1196	6	294

We compare our proposed method with two conventional feature selection algorithms and a well-known EA-based multi-objective algorithm. The two conventional methods are the ReliefF method (RF-BR) proposed in the literature^5^, and the mutual information method (MI-PPT) proposed in the literature^7^. The evolutionary multi-objective algorithm is NSGA-II^30^. It is one of the most popular multi-objective evolutionary algorithms. The main principle of NSGA-II is the application of the fast no-dominated sorting technique and the diversity preservation strategy. The idea of NSGA-II has been used to deal with single-label feature selection problems^31, 32^.

In the proposed algorithm and NSGA-II, the size of swarm or population is set to 20, and the fitness evaluation times as 2000 for all the test problems. In NSGA-II, the representation of each individual is the same as the proposed algorithm, the mutation rate is *1*/*D* and the crossover probability is 0.9. The ML-KNN^21^ is used as the classifier in this paper.

### Comparison on the smallest Hamming loss

Taking the three datasets, Emotions, Yeast and Scene as examples, this section evaluates the proposed algorithm's performance on finding extreme solution with the smallest Hloss value. Table 2 shows the best Hloss value and the number of selected features obtained by the two conventional methods, RF-BR and MI-PPT, NSGA-II, and the proposed algorithm. Additionally, the bold data are the best values among these algorithms.

**Table 2:** Solutions with the smallest Hloss value found by the four comparison algorithms.

Datasets	Proposed algorithm		MI-PPT		RF-BR		NSGA-II	
	Hamming loss	Number of features	Hamming loss	Number of features	Hamming loss	Number of features	Hamming loss	Number offeatures
Emotions	**0.178**	27	0.229	43	0.220	**17**	0.183	47
Yeast	**0.193**	45	0.194	91	0.240	**41**	0.196	56
Scene	**0.088**	164	0.092	278	0.120	**56**	0.092	123

As shown in Table 2, the proposed algorithm performs better than RF-BR, MI-PPT and NSGA-II in searching for those solutions with the smallest Hloss value. In details: (1) the RF-BR algorithm has the best solutions with respect to the number of features, but our proposed algorithm shows the best performance with respect to Hamming loss. For example, for the dataset Scene, the best Hloss value of our algorithm reduces by 4.2% compared with RF-BR; (2) Compared with MI-PPT, the proposed algorithm has a small Hamming loss with small number of features when classifying Emotions. For the rest data sets, Scene and Yeast, the proposed algorithm not only reduces the number of features significantly, but also improves the minimum of Hamming loss; (3) Compared with NSGA-II, the proposed algorithm has still the smallest Hloss for all the three data sets. Especially, for Emotions and Yeast, it shows the best capability to remove irrelevant or redundant features.

### Assessment on the parallel search capability

**Figure 3:**
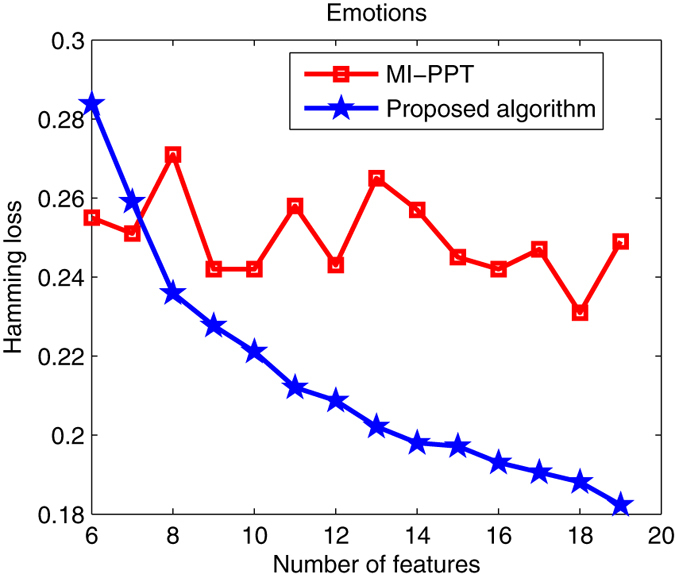
Pareto optimal sets found by MI-PPT and our algorithm on Emotions.

**Figure 4:**
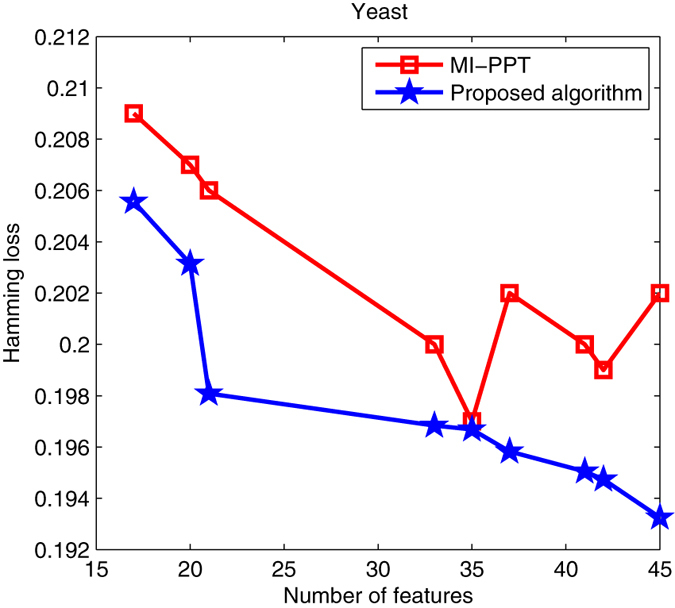
Pareto optimal sets found by MI-PPT and our algorithm on Yeast.

**Figure 5:**
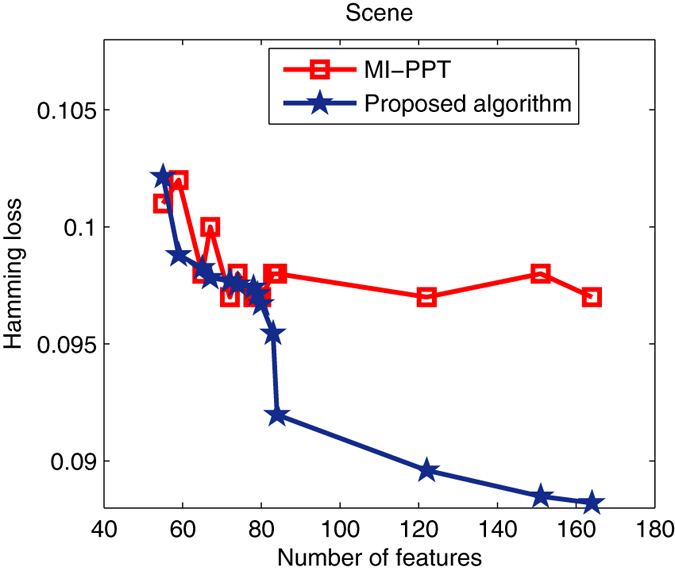
Pareto optimal sets found by MI-PPT and our algorithm on Scene.

This subsection tests the parallel search performance of our algorithm. Classical MI-PPT method is often inefficient for solving multi-objective feature selection, because it can only find a few of optimal solutions in a single run, suggesting that it has to run several times to achieve a good Pareto set. The proposed PSO-based algorithm in this paper, on the other hand, is a population-based, metaheuristic method with the ability to search for multiple Pareto solutions in one run. Taking Emotions, Yeast and Scene as examples, Figs 3, 4 and 5 shows Pareto optimal sets obtained by MI-PPT and our algorithm when tackling the three data sets.

For Emotions, we can see from Fig. 3 that: when 6 and 7 features are selected only, the proposed algorithm obtains worse results than MI-PPT in terms of the Hamming loss. However, the Hamming loss values of the proposed algorithm drop fiercely from about 23.6% to 18% when the number of features increases from 8 to 19. Contrarily, MI-PPT shows an unstable curve, where its Hamming loss values move around 24.5% up and down as the number of features increases. When 18 features is selected, MI-PPT finds the best Hamming loss, 23.1%, which is obviously bigger than the value obtained by the proposed algorithm, 18.8%. The proposed algorithm has the best Hamming loss 18.23% with 19 features.

For Yeast, we can see from Fig. 4 that the Hamming loss values of the proposed algorithm drop fiercely from about 20.6% to 19.3% when the number of features increases from 17 to 45. Like Emotions, MI-PPT shows also an unstable curve. When 35 features are selected, MI-PPT finds the best Hamming loss 19.7%, which is still slightly bigger than the value obtained by the proposed algorithm, 19.67%. Our proposed algorithm finds the best Hamming loss value when 45 features are selected, which get a 0.9% lower error rate compared with MI-PPT.

Figure 5 shows the solutions on optimizing Scene. We can see that: when less than 80 features are selected, MI-PPT has more close results as the proposed algorithm; but it can’t obviously improve the Hamming loss by increasing the number of features. The proposed algorithm achieves clearly better results than MI-PPT when more than 80 features are selected. It finds the best Hamming loss value, 8.8%, when 164 features are selected, which gets an about 0.8% lower loss value compared with MI-PPT.

Furthermore, the set coverage (SC) measure^33^ is employed to compare the domination degree between different algorithms. Taking algorithms Z1 and Z2 as an example, SC(Z1, Z2) = 1 represents that each solution of Z2 is dominated by or equal to at least one solution of Z1, indicating that the Pareto solutions founded by Z1 is better than those obtained by Z2.

Table 3 shows the average SC values of the proposed algorithm and M-PPY. It reports that the proposed algorithm has the best performance with respect to the SC metric for all the three datasets. In details, for Emotions, solutions obtained by the proposed algorithm dominate 85.71% solutions obtained by MI-PPT; in contrast, the proportion that MI-PPT dominates the proposed algorithm is 14.29%. For Yeast, the proportion that MI-PPT dominates the proposed algorithm is 0, but our proposed algorithm dominates all the solutions of MI-PPT. For Scene, the proportion that MI-PPT dominates the proposed algorithm still is 64.29. Overall, due to good parallel search capability, the proposed algorithm can find a set of optimal solutions, which is better than those obtained by MI-PPT.

**Table 3:** The average set coverage values of the two algorithms.

Datasets	(Proposed algorithm, MI-PPT)	(MI-PPT, Proposed algorithm)
Emotions	0.8571	0.1429
Yeast	1.0	0
Scene	0.6429	0.4286

### Assessment on the multi-objective performance

The proposed algorithm is compared with the popular algorithm NSGA-II to test its multi-objective performance. Herein, hyper-volume metric (HV)^33^ is introduced to estimate a multi-objective algorithm, because it can simultaneously estimate the distribution and the convergence of a solution set. The better the diversity and/or convergence of a solution set are, the higher the HV value of this set is.

The two algorithms both are run 30 times for all the six datasets, statistical results of the two algorithms are showed in Table 4. Furthermore, the paired *t*-tests at the significant level of 0.05 (α = 0.05) is utilized to test the significance of results with respect to the HV metric. In this table, ‘Y+’ indicates that the proposed algorithm is significantly better than NSGA-II. As Table 4 shows, the proposed algorithm has better HV values for all the six data sets, and its multi-objective performance is significantly better than NSGA-II as their *t*-test results.

**Table 4:** The average HV values obtained by the two algorithms on the six datasets.

Data sets	Proposed algorithm	NSGA-II	t-test
Flags	0.738/0.076	0.592/0.063	Y+
CAL500	0.798/0.077	0.595/0.022	Y+
Emotions	0.766/0.029	0.553/0.036	Y+
Yeast	0.709/0.036	0.508/0.011	Y+
Birds	0.859/0.043	0.580/0.015	Y+
Scene	0.756/0.021	0.560/0.014	Y+

**Figure 6:**
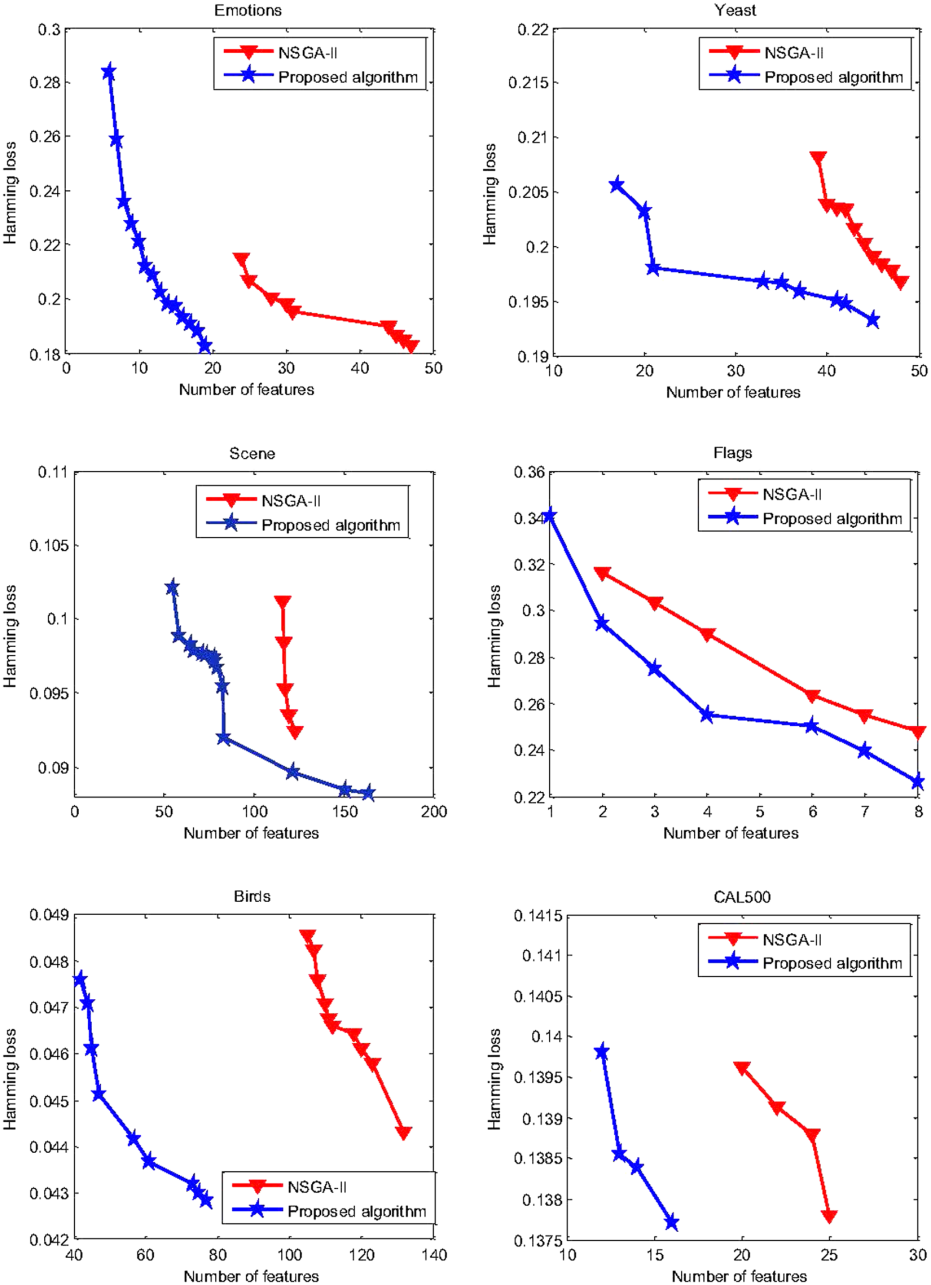
Solutions obtained by our algorithm and NSGA-II on six datasets.

Furthermore, Fig. 6 shows optimal solution sets obtained by the two algorithms on the six datasets, for highlighting their search capability. Clearly, the proposed algorithm shows better convergence than NSGA-II, where each solution of NSGA-II is dominated by at least one of the proposed algorithm. So we consider that the proposed algorithm outperforms NSGA-II in terms of the multi-objective performance.

### Analyses of the key operators

In this experiment we perform an extensive analysis on the two key operators, i.e., the adaptive mutation and the local learning strategy. For the sake of simplicity, our proposed algorithm is denoted as MPSOFS in this experiment. Four compared algorithms are designed. The first one is the proposed algorithm which deletes the local learning strategy (LLS), denoted as MPSOFS/LLS; the second one is the proposed algorithm without mutation, denoted as MPSOFS/M; the third one is the proposed algorithm with the nonuniform mutation (NM) proposed in ref. 4, denoted as MPSOFS-NM; the last one is the proposed algorithm with the Pareto rank based mutation (PRM) proposed in ref. 34, denoted as MPSOFS-PRM. In the PRM, the mutation probability of a particle is determined by both the current iteration times and the fitness rank of the particle. For details, please see the literature^4, 34^. The HV metric is selected to estimate these algorithms. The datasets, Emotions, Yeast and Scene, are used to analyze the two key operators.

**Figure 7:**
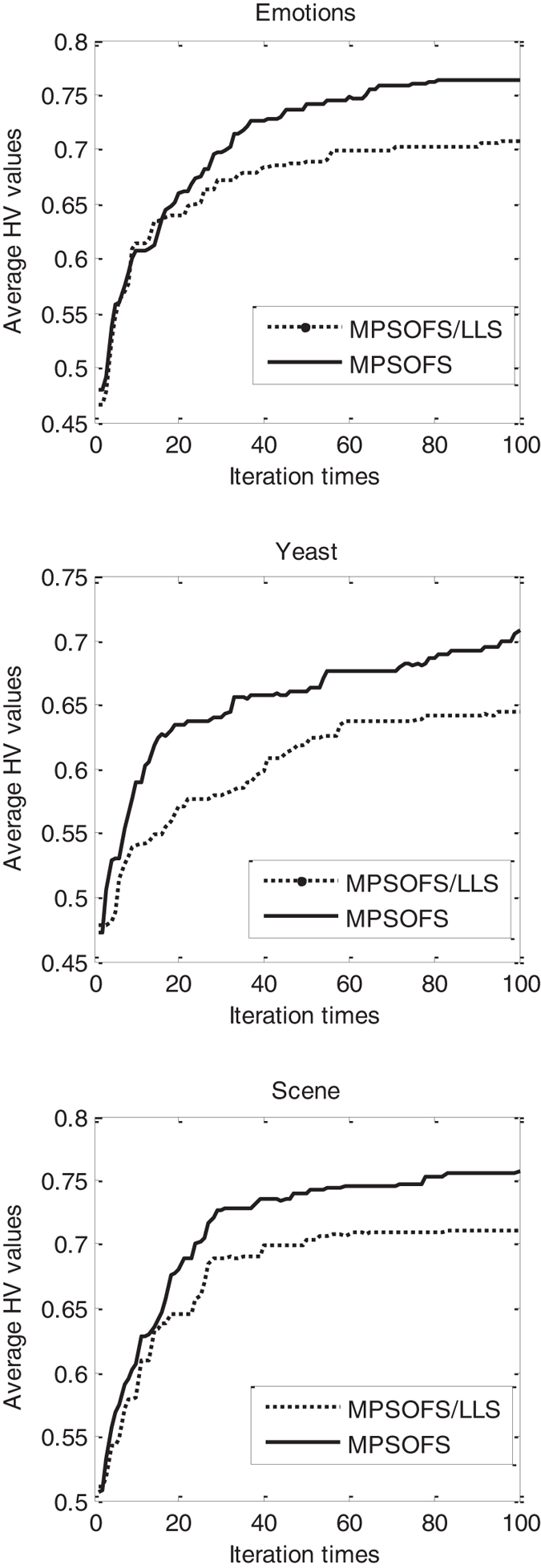
The curve of HV values with respect to the iterations obtained by MPSOFS and MPSOFS/LLS.

First, we compare the proposed algorithm MPSOFS to MPSOFS/LLS, for observing the effect of the local learning strategy. Figure 7 shows the curve of HV values with respect to the iteration times obtained by MPSOFS and MPSOFS/LLS. It can be seen from Fig. 7 that for all the three datasets, MPSOFS shows better convergence than MPSOFS/LLS with respect to the HV value. Taking Emotions as an example, MPSOFS has the best HV value 76.4% at the100-th iterations, but MPSOFS/LLS has its best HV value 70.7% at the100-th iterations, which is almost 6 percentage points lower than MPSOFS. This indicates that the effect of the local learning strategy on improving the performance of MPSOFS is visible.

**Figure 8:**
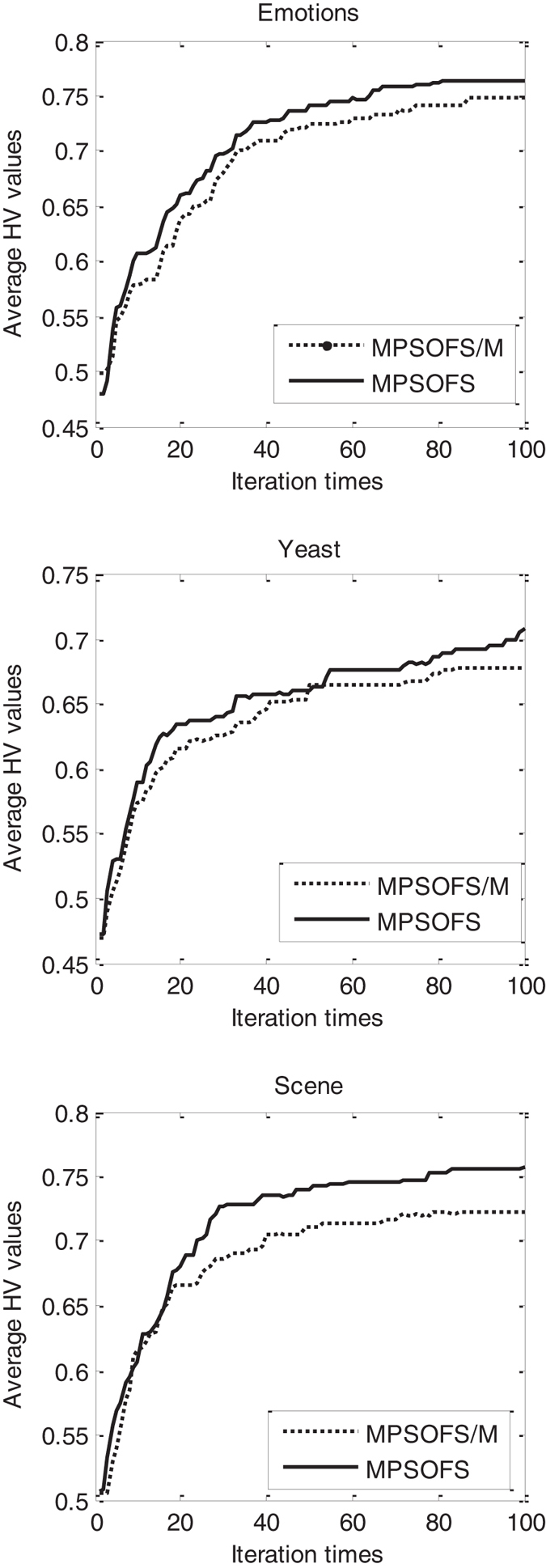
The curve of HV values with respect to the iterations obtained by MPSOFS and MPSOFS/M.

Second, we compare the proposed algorithm MPSOFS to MPSOFS/M, for observing the effect of the adaptive mutation on the proposed algorithm. Figure 8 shows the curve of HV value with respect to the iteration times obtained by the two compared algorithms. It reports that for the three datasets, the mutation-based algorithm MPSOFS shows better convergence curves than MPSOFS/M with respect to the HV metric. Moreover, at the end of algorithm, the best HV value obtained by MPSOFS is also obviously higher than that of MPSOFS/M. Taking Yeast as an example, the best HV values of MPSOFS and MPSOFS/M are 70.9% and 67.1% at the100-th iterations, respectively. This indicates that the mutation is important on improving the performance of MPSOFS.

Furthermore, we compare the three mutation-based algorithms, MPSOFS, MPSOFS-NM and MPSOFS-PRM, for observing the effectiveness of the adaptive mutation. Table 5 shows statistical results of HV obtained by the three algorithms for the datasets Emotions, Yeast and Scene. As Table 5 reports, for the dataset Emotions with 72 features, MPSOFS, MPSOFS-NM and MPSOFS-PRM have similar results with respect to the average HV value. However, for the datasets Yeast and Scene with more 100 features, the average HV values of MPSOFS-NM are obviously smaller than that of MPSOFS and MPSOFS-PRM. Taking the dataset Yeast as example, the average HV values of MPSOFS, MPSOFS-NM and MPSOFS-PRM are 70.9%, 68.6% and 71.0%, respectively. On the other hand, MPSOFS show a close performance to MPSOFS-PRM with respect to the HV metric for the three datasets. Here, the average HV values of MPSOFS are slightly higher than that of MPSOFS-PRM for Emotions and Scene, while MPSOFS-PRM has the best average HV value for Yeast, which is slightly higher than MPSOFS. However, since a Pareto rank relationship between all the particles needs be built every time the swarm implements the PRM mutation, the run time of MPSOFS-PRM is higher than MPSOFS for all the three datasets. For example, the ratio of run time between MPSOFS and MPSOFS-PRM on tacking the dataset Emotions is about 1:1.07. Thus our proposed mutation is highly competitive compared to the NM mutation and the PRM mutation.

**Table 5:** The average HV values obtained by MPSOFS, MPSOFS-NM and MPSOFS-PRM on the three datasets.

Data sets	MPSOFS	MPSOFS-NM	MPSOFS-PRM
Emotions	0.766/0.029	0.762/0.025	0.764/0.027
Yeast	0.709/0.036	0.686/0.031	0.710/0.039
Scene	0.756/0.021	0.738/0.020	0.754/0.024

## Discussion

In this paper, a PSO-based multi-objective multi-label feature selection algorithm has been presented. In this algorithm, the probability-based encoding was introduced to transform a discrete feature selection problem into a continuous one suitable for PSO. The idea of non-dominated comparison, as well as the crowding distance, was used to prune the archive. And, the adaptive uniform mutation, combined with the local learning strategy, enhanced significantly search capability of the proposed algorithm.

The proposed feature selection algorithm is examined and compared with two traditional methods (RF-BR and MI-PPT) and the popular NSGA-II approach. According to their experiments, we can find: (1) The proposed algorithm has a good capability in searching for the extreme solution with the best Hloss value; (2) The proposed algorithm found a set of feature subsets with small Hamming loss in one run; (3) The newly presented operators, together with the established operators, make the proposed algorithm has a good capability in exploration than NSGA-II. In the future, we will investigate meta-heuristic-based feature selection approaches for new feature selection problems, such as cost-based feature selection.
